# Hyperintense Perilesional Edema on T1-Weighted Imaging in Cavernoma: A Case Report

**DOI:** 10.7759/cureus.51454

**Published:** 2024-01-01

**Authors:** Ibrahim A Alturki, Tareq A Alluhidan, Abdulrahman A Alturki, Faisal S Alzahrani, Nawaf A Alluwaymi

**Affiliations:** 1 Radiology Department, King Khalid University Hospital, Riyadh, SAU; 2 General Practice, The Ministry of Health, Riyadh, SAU

**Keywords:** neuro radiology, mri findings, vascular anomaly, perilesional edema, cerebral cavernoma

## Abstract

Cavernoma, also called a cavernous malformation, is a vascular malformation that happens during development. It tends to look like a berry-shaped lesion. In cerebral hemorrhagic cavernous malformations (cavernoma), T1-weighted imaging that shows hyperintense perilesional edema in brain masses is an unusual radiological finding. This sign's association with cavernoma is gaining prominence.

We present the case of a 35-year-old female patient without significant medical history who reported a seven-day history of left-side weakness that began in the upper limb, progressed to the lower limb, and was associated with nausea. The non-contrast T1- T1-weighted images displayed a gradient of hyperintense content of the lesion with surrounding relatively hyperintense perilesional edema. The patient consequently underwent surgery to evacuate the hematoma and excise the lesion, which went uneventfully.

## Introduction

Cavernoma is a vascular malformation that occurs during development and is also referred to as a cavernous malformation. It tends to look like a berry-shaped lesion with multiple lobes and bleeding at different stages. Hemorrhage is a common complication of cavernoma; in 30% of cases, it is the first sign of the disease [1]. In cerebral hemorrhagic cavernous malformations (CCM, cavernoma), T1-weighted imaging (T1WI) that shows hyperintense perilesional edema in brain masses is an unusual radiological finding. This sign's association with cavernoma is gaining prominence [2].

Cavernous malformations (CMs) or cavernomas are infrequent, radiographically hidden lesions that develop in the central nervous system, with an estimated 0.5% (0.4-0.6%) occurrence in the general population. They are the second most prevalent kind of cerebrovascular lesion, accounting for 10% to 15% of all cerebral vascular abnormalities. The supratentorial areas, basal ganglia, brain stem, cerebellopontine angle, and cerebellar hemispheres are frequent locations for intracranial CMs. The incidence of these disorders in the brainstem ranges from 4% to 35%. These lesions are generally surrounded by hemosiderin and gliosis, but no brain parenchyma is normally identified within the lesion [3].

The high signal intensity found in brain lesions at T1-weighted magnetic resonance (MR) imaging is caused by a variety of chemicals, including methemoglobin, melanin, lipid, protein, calcium, iron, copper, and manganese [4]. The T1WI, or the "spin-lattice" relaxation time, is one of the basic pulse sequences in MRI, which shows how the T1 relaxation times of different tissues are different [5]. A T1WI depends on how the net magnetization vector (NMV) of a tissue relaxes along its length. Basically, a radiofrequency (RF) pulse moves spins that are aligned in an external field (B0) into the transverse plane. Then, they slide back toward B0, where they were before [5].

This case signifies the importance of being vigilant while assessing imaging for patients suspected of having a brain tumor. When cavernous malformations are suspected, this unusual magnetic resonance (MR) characteristic of cavernous angiomas, which contrasts with the typical reticulated "popcorn ball" sign, should be addressed more thoroughly.

This article was previously presented as a poster at the 5th Research Day at Imam Muhammad Ibn Saud University on May 27, 2023.

## Case presentation

A 35-year-old female patient without a significant medical history presented to the emergency department complaining of a seven-day history of left-side weakness that began gradually from an upper limp and then involved a lower limp, with gait changes and falling suddenly due to weakness. The weakness was accompanied by nausea. The patient denied experiencing any loss of consciousness, seizures, or visual changes. During the physical examination, the patient was aware and oriented to time, place, and person. Language and attention exams were both intact. Limbs power was 4/5 on the left side and 5/5 on the right. The patient's tone was normal, and she exhibited bilateral Hoffman's sign. At the time, the differential diagnosis could be anything from stroke to thrombosed arteriovenous malformations or aneurysms to any other type of brain tumor.

**Figure 1:**
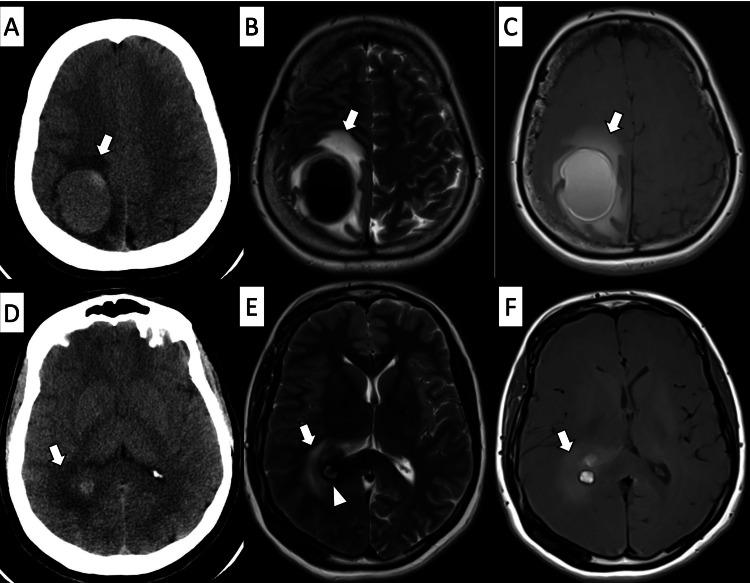
CT and MRI scan showing hyperintense perilesional edema A and D are selective images of an unenhanced CT scan of the brain at the level of the centrum semiovale and posterior horn of the right lateral ventricle, respectively. They exhibit an iso- to hyper-dense, well-defined lesion with surrounding vasogenic edema (arrow) within the right parietal lobe. B and E are T2-weighted images at the same mentioned levels, displaying a hypointense lesion superiorly and hyperintense content inferiorly with a hypointense rim (arrowhead on E) and surrounding hyperintense vasogenic edema (arrow). C and F show the same lesion on T1-weighted images without contrast, demonstrating a gradient of hyperintense content with a hypointense rim and hyperintense perilesional edema (arrow).

Imaging studies were crucially important for further assessment. A CT and MRI of the brain were obtained, which showed a gradient of hyperintense content of the lesion with surrounding relatively hyperintense perilesional edema (Figure 1). The unenhanced CT scan of the brain showed a well-defined iso- to hyper-dense lesion with surrounding vasogenic edema within the right parietal lobe, causing mild mass effect upon the adjacent sulci and posterior horn of the right lateral ventricle. However, there was no midline shift, hydrocephalus, or brain herniation. On the T2-weighted MRI images, the lesion exhibited a fluid-fluid level with predominantly hypointense content. On the other hand, the non-contrast T1-weighted images displayed a gradient of hyperintense content of the lesion with surrounding relatively hyperintense perilesional edema. The most inferior aspect of the lesion exhibited hyperintense content with a rim of hypointensity, featuring a popcorn appearance (Figure 1).

**Figure 2:**
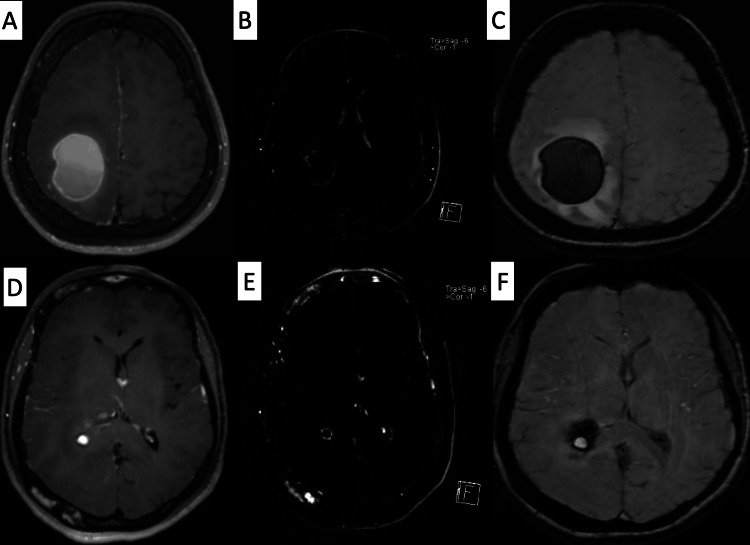
MRI T1 images post contrast A and D are T1-weighted images post-contrast with subtraction images (B and E) at two different levels of the same lesion showing a thin rim of enhancement. On susceptibility-weighted images (C and F), the lesion contained a susceptibly effect and a relatively higher rim of susceptibility.

Similarly, the susceptibility-weighted images demonstrated a susceptibility effect. The post-contrast images showed a margin of thin enhancement, best seen in subtraction images (Figure 2).

**Figure 3:**
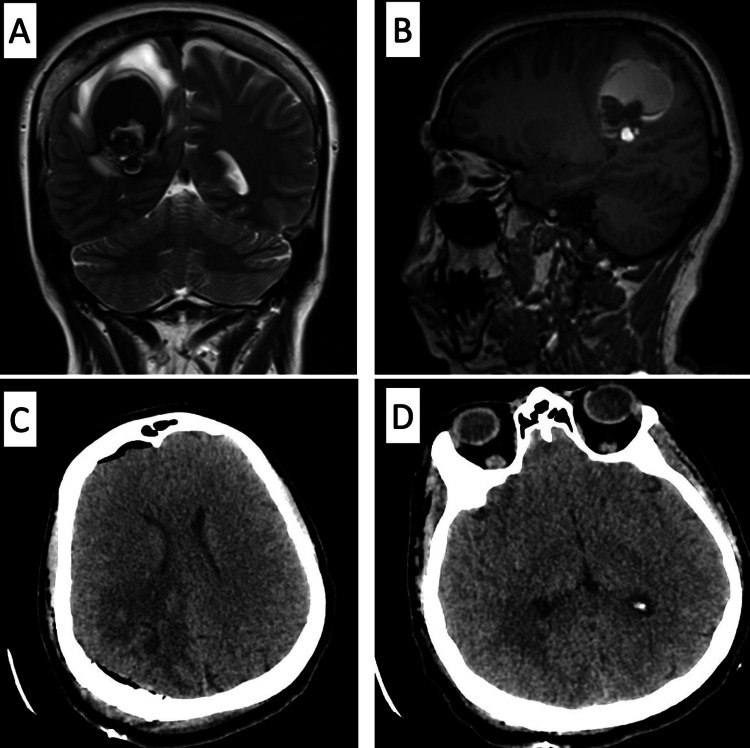
CT and MRI scans The lesion showed a fluid-fluid level, which is best displayed by the coronal T2-weighted image (A) and sagittal T1-weighted image (B). Status post excision of the lesion with immediate post-operative changes are noted on an unenhanced CT scan of the brain (C and D).

Coronal and sagittal images of T2 and T1 weighted sequences (Figure 3A-3B), displayed the extent of the lesion and the fluid-fluid level. The constellation of the findings were most consistent with a cavernoma. The post-operative CT scan showed the expected immediate post-operative changes without complications or residual lesion (Figure 3C-3D).

Surgery was planned to follow these findings. The patient had an uneventful right parietal craniotomy to evacuate the hematoma and excise the lesion. A right parietal subacute hematoma with a mulberry-looking lesion was found intraoperatively. Following surgery and anesthetic recovery, the patient was completely awake, following directions, communicating, stable, and transferred to the post-anesthesia care unit (PACU). Following that, the patient was transferred from the PACU to the ward two hours after surgery and discharged on day five post-op. 

Post-operative central nervous system (CNS) findings were significant for midline shift interval change, measuring now 0.5 cm compared to 0.7 cm previously. There was no evidence of uncal or tonsillar herniation. Fibrous dysplasia was represented by unchangeable widespread sclerotic and expansile bone lesions. There was no acute well-established territorial infarction in the rest of the brain parenchyma. There was no acute intra/extra-axial bleeding. The structures of the posterior fossa were unremarkable. Paranasal sinuses and mastoid air cells were well aerated. The impression was that the patient was in good condition following the right parietal craniotomy, with the predicted post-operative changes and better midline shift described above. There was no acute intracranial injury.

In terms of wound status and follow-up plan, the wound was clean and dry; she was discharged on day five post-op with analgesia and outpatient clinic follow-up after one week. She came to the clinic doing well, with weakness improved on the left side, with an MRI planned in two months. MRI was done after two months, which showed complete resection of the right parietal cavernoma with no signs of residual. She was seen again and doing well with improvement in weakness. The patient was seen doing well with on/off discomfort at the surgery site at the six-month follow-up, with a planned one-year follow-up.

## Discussion

The most significant diagnostic approach for detecting cavernoma is MR imaging, which typically gives an extremely distinctive appearance. Cavernoma typically has a mixed signal intensity core, a reticulated "popcorn ball" look, and a "T2 blooming sign" caused by a low signal intensity hemosiderin rim that fully surrounds the lesion [1]. The atypical MR features of cavernous angiomas include variable or strong enhancement. Susceptibility-weighted imaging, such as a T2* gradient-echo picture, is more effective in detecting hemosiderin deposits and diagnosing cavernoma. On T2-weighted images, the characteristic MR signals of a "popcorn ball" look and "T2 blooming sign" have been shown to be present in roughly 50-67% of cavernoma [1].

Most symptomatic individuals are between the ages of 40 and 60, and the majority present with a solitary lesion. Multiple lesions may be inherited; therefore, family members should undergo screening. In addition, cavernous anomalies and capillary telangiectasias are common following radiation to the brain. On neuroimaging, around 40% (range: 20-50%) of cavernous malformations are detected incidentally [6]. Cavernous malformations are histologically formed of a "mulberry-like" cluster of hyalinized dilated thin-walled capillaries surrounded with hemosiderin. To varying degrees, these veins are thrombosed. Unlike arteriovenous malformations (AVMs), there is no normal brain between the interstices of these lesions [6].

Sometimes, cavernous malformations with acute hemorrhage and significant perilesional edema might mimic other cerebral lesions. It has been shown that T1WI hyperintensity in perilesional edema is highly suggestive of cavernous malformations. Occasionally, a cavernoma with acute hemorrhage and significant perilesional edema might mimic different cerebral lesions. The literature described that T1WI hyperintensity in perilesional edema is advantageous in narrowing the differential diagnosis, emphasizing cavernous malformations. However, other intra-axial lesions, including malignant and benign lesions, were associated with perilesional hyperintensity on T1WI. Furthermore, a patient with a non-neoplastic intraparenchymal hemorrhage also can exhibit perilesional hyperintensity [1].

According to studies, intraparenchymal hemorrhage increases the expression of inflammatory markers in the brain, resulting in serum protein penetrating the surrounding white matter and edema. Also, studies have shown that the edema around an intraparenchymal hematoma develops within hours of the occurrence and reaches its peak between 10 and 20 days afterward [7].

## Conclusions

This case emphasizes the need for vigilance in evaluating imaging for patients suspected of brain tumor. This atypical magnetic resonance (MR) feature of cavernous angiomas, which opposes the typical reticulated "popcorn ball" sign, should, therefore, be addressed more when cavernous malformations are suspected. More research literature on hyperintense perilesional edema on T1 and its correlation with cavernous malformations will enhance our understanding of this case, improve the quality of patient care, and help expedite the early diagnosis. Early surgical intervention is crucial for better outcomes, and thus, more research literature on this topic is needed. Ultimately, the combination of MRI findings featuring cavernoma with emphasis on the perilesional hyperintensity on T1WI, as well as the clinical data, helped identify a cerebral cavernoma.

## References

[1] Yun TJ, Na DG, Kwon BJ, Rho HG, Park SH, Suh YL, Chang KH (2008). A T1 hyperintense perilesional signal aids in the differentiation of a cavernous angioma from other hemorrhagic masses. Am J Neuroradiol.

[2] Sarbu N, Pujol T, Oleaga L (2016). Hyperintense perilesional edema in the brain on T1-weighted images: cavernous malformation or metastatic melanoma? Three case reports and literature review. Neuroradiol J.

[3] Rajagopal N, Kawase T, Mohammad AA, Seng LB, Yamada Y, Kato Y (2019). Timing of surgery and surgical strategies in symptomatic brainstem cavernomas: review of the literature. Asian J Neurosurg.

[4] Ginat DT, Meyers SP (2012). Intracranial lesions with high signal intensity on T1-weighted MR images: differential diagnosis. Radiographics.

[5] Baba Y, Jones J (2009). T1 weighted image. Radiopaedia.

[6] D'Souza D, Machang'a K, Ranchod A (2008). Cerebral cavernous venous malformation. Radiopaedia.

[7] Cortés Vela JJ, Concepción Aramendía L, Ballenilla Marco F, Gallego León JI, González-Spínola San Gil J (2012). Cerebral cavernous malformations: spectrum of neuroradiological findings. Radiología.

